# BTG2 and SerpinB5, a novel gene pair to evaluate the prognosis of lung adenocarcinoma

**DOI:** 10.3389/fimmu.2023.1098700

**Published:** 2023-03-17

**Authors:** Wanting Yang, Chunli Wei, Jingliang Cheng, Ran Ding, Yan Li, Yonghua Wang, Yinfeng Yang, Jinghui Wang

**Affiliations:** ^1^ School of Integrated Chinese and Western Medicine, Anhui University of Chinese Medicine, Hefei, Anhui, China; ^2^ Key Laboratory of Epigenetics and Oncology, the Research Center for Preclinical Medicine, Southwest Medical University, Luzhou, Sichuan, China; ^3^ School of Medical Informatics Engineering, Anhui University of Chinese Medicine, Hefei, Anhui, China; ^4^ Key Laboratory of Industrial Ecology and Environmental Engineering (MOE), Faculty of Chemical, Environmental and Biological Science and Technology, Dalian University of Technology, Dalian, Liaoning, China; ^5^ College of Life Sciences, Northwest University, Shaanxi, China

**Keywords:** BTG2, SerpinB5, LUAD, immunotherapeutic, tumor microenvironment

## Abstract

**Introduction:**

Lung adenocarcinoma (LUAD), as the most frequent pathological subtype of non−small cell lung cancer, is often characterized by poor prognosis and low 5-year survival rate. Exploriton of new biomarkers and accurate molecular mechanisms for effectively predicting the prognosis of LUAD patients is still necessary. Presently, BTG2 and SerpinB5, which play important roles in tumors, are studied as a gene pair for the first time with the aim of exploring whether they can be used as potential prognostic markers.

**Methods:**

Using the bioinformatics method to explore whether BTG2 and SerpinB5 can become independent prognostic factors, and explore their clinical application value and whether they can be used as immunotherapeutic markers. In addition, we also verify the conclusions obtained from external datasets, molecular docking, and SqRT-PCR.

**Results:**

The results show that compared with normal lung tissue, BTG2 expression level was down-regulated and SerpinB5 was up-regulated in LUAD. Additionally, Kaplan–Meier survival analysis demonstrate that the prognosis of low expression level of BTG2 was poor, and that of high expression level of SerpinB5 was poor, suggesting that both of them can be used as independent prognostic factors. Moreover, the prognosis models of the two genes were constructed respectively in this study, and their prediction effect was verified by external data. Besides, ESTIMATE algorithm reveals the relationship between this gene pair and the immune microenvironment. Furthermore, patients with a high expression level of BTG2 and a low expression level of SerpinB5 have higher immunophenoscore for CTLA-4 and PD-1 inhibitors than patients with a low expression level of BTG2 and a high expression level of SerpinB5, indicating that such patients have a more obvious effect of immunotherapy.

**Discussion:**

Collectively, all the results demonstrate that BTG2 and SerpinB5 might serve as potential prognostic biomarkers and novel therapeutic targets for LUAD.

## Introduction

Lung cancer is the third most common cancer in the world and the leading cause of cancer death worldwide. According to the histological classification of tumors, lung cancer can be divided into two types: small cell lung cancer, accounting for 15% of all lung cancer, and non-small cell lung cancer (NSCLC), accounting for about 85% of all lung cancer (1, 2). Among them, NSCLC can be divided into several histological subtypes: lung adenocarcinoma (LUAD), adenosquamous cell carcinoma, squamous cell carcinoma (LUSC) and large cell carcinoma (LCC) (3), in which LUAD is by far the most common subtype of NSCLC. The main reason for the high mortality of LUAD was the lack of early diagnosis methods that would find tumorigenesis at an early stage. So tumorigenesis can’t be found in time in the early stage of cancer, leading to the diagnosis of most patients in the middle and late stages (4). At the same time, the tumor was prone to invasion and metastasis, resulting in poor curative effect (5).

Cisplatin is currently the first-line drug for the treatment of lung cancer, but the clinical application is limited due to drug resistance (6, 7). However, cisplatin is often used in combination with other drugs in the process of clinical medication. Although cisplatin has a strong tolerance to lung cancer, its basic pharmacological effect against lung cancer is still worthy of further study (8, 9). In this study, we use bioinformatics technology to predict the core targets during the development of LUAD, taking cisplatin as the main drug for the treatment of lung cancer to find the targets that could be used as prognostic markers. Through bioinformatics study of gene expression changes in LUAD patients after being treated by cisplatin that the data was downloaded from GEO dataset, it was found that cisplatin could regulate the abnormal decrease or increase of gene expression level of BTG2 and SerpinB5 in Lung cancer cells, and these two genes were related to the overall survival (OS) of the LUAD patients. Additionally, from the correlation of gene expression, which was calculated by Pearson’s correlation test, it was found that there was a negative correlation between BTG2 and SerpinB5. Relevant studies have also found that both of them were related to p53 (10, 11). The expression level of BTG2 was related to the SerpinB5’, and the two genes could interact through p53. Therefore, we took BTG2 and SerpinB5 as a new gene pair to study their clinical prognostic value.

Actually, BTG2 was the first gene found in the BTG/TOB gene family, which was involved in biological functions such as cell proliferation and differentiation, cell cycle regulation, and DNA damage repair (12). A large number of studies have shown that the expression level of BTG2 in tumors was closely related to the biological characteristics of tumors (12–15). The BTG2 was considered to be a tumor suppressor gene, and the expression level was significantly reduced or even not expressed in liver cancer, bladder cancer, breast cancer, ovarian cancer and other tumors (16). With respect to SerpinB5, it was one of the members of the serine protease inhibitor (Serpin) family, belonging to non-inhibitory subpins (17). SerpinB5 was expressed in normal breast epithelial cells, skin, prostate, testis, lung, tongue, intestine and thymus, but the expression level was abnormally lower in a variety of malignant tumors compared with the expression level in normal tissue. Previous studies have shown that SerpinB5 can inhibit the occurrence and development of tumors, including promoting tumor cell apoptosis, inhibiting tumor angiogenesis, and inhibiting tumor metastasis (18–20).

Presently, we performed a series of bioinformatics analyses on the gene expression level of BTG2 and SerpinB5 in LUAD, including transcriptional analysis, co-expression analysis, functional annotation enrichment analysis, protein-protein interaction (PPI) analysis, survival analysis, and constructed prognosis models. The increased levels of SerpinB5 and decreased BTG2 expression were observed in LUAD. Both a high expression level of SerpinB5 and a low expression level of BTG2 were associated with poor OS in LUAD. In addition, the expression level of BTG2 and SerpinB5 were related to macrophages in the immune microenvironment, which may be an important reason why these two genes can affect the immune microenvironment. Finally, we verified our research content through many methods, including external datasets, molecular docking, immunohistochemistry, and experiment which would make our findings more reliable. In our article, these two genes were studied together for the first time. We studied whether this gene pair could be a potential tumor prognostic marker and its potential mechanism. All these findings provide new insights for improving the prognosis of patients and may may promote the discovery and application of prognostic markers of LUAD.

## Materials and methods

### Data sources

The gene expression matrix of patients with LUAD samples was downloaded from the Gene Expression Omnibus (GEO) website (https://www.ncbi.nlm.nih.gov/), including GSE73302 datasets. The corresponding probe set GPL5175 of GSE73302 dataset was obtained from GEO website. Gene expression profile data of LUAD patients were downloaded from the TCGA database (https://portal.gdc.cancer.gov/repository), which included 59 samples of normal lung tissue and 539 LUAD tissues (Workflow Type: STAR-Counts). Four groups of samples were in GSE73302 dataset, including A549 cell samples that were not treated with cisplatin and cultured for 24 and 48 hours respectively as the experimental control group, and A549 cell samples treated with cisplatin for 24 and 48 hours respectively as the experimental group, each group repeated three times. Therefore, a total of 12 samples were analyzed in GSE73302 dataset. The pan-cancer analysis of genes in 33 kinds of cancers was obtained through Sangerbox (http://sangerbox.com/tool.html) database. Data on pan-cancer analysis in the Sangerbox were downloaded from UCSC XENA, which was from TCGA database and GTXs and the expression value was converted into Log2 (x+0.001).

### Identification of DEGs in LUAD after treated with cisplatin

In order to obtain the differential expression genes (DEGs), the gene expression data need to be preprocessed, including the data correction and log2 (x+1) transformation. First, we corrected the gene expression data through the normalized BetweenArrays function of the “limma” package of R (4.2.0) and then calculated the log2 (x+1) of the corrected data. DEGs in GSE73302 were obtained by using the “limma” package. The gene expression level of DEGs in GSE73302 was visually displayed by heatmap and the volcano plot, which were drawn through the “ggplot2” package. The y-axis of the volcano plot is log2 fold change (log2FC) and the fold change represents the differential expression multiple. The expression of these genes that were increasing or decreasing can be judged by the positive and negative value of log2 fold change in the volcano plot.

The DEGs in normal tissues and tumor tissues were obtained by using the “limma” package. The screening criteria of DEGs were P < 0.05 and |log FC| ≥1.0. In order to obtain DEGs in tumor tissues after cisplatin interference, the overlapping DEGs of two gene expression profiles were obtained through the “Venn” package.

### Protein-protein interaction network

In order to explore the interaction between DEGs, a PPI network was constructed. We obtained the gene interaction relationship among 17 DEGs through the online database STRING (https://cn.string-db.org/) and constructed a PPI network through Cytoscape (3.8.0). Meanwhile, the correlations of gene expression between the 17 DEGs were calculated by Pearson’s correlation analysis and displayed by a heatmap.

### Survival analysis of DEGs

To evaluate whether mRNA levels of DEGs affected the prognosis of LUAD, the correlation between the expression level of 17 DEGs and median OS were analyzed using the GEPIA database (http://gepia.cancer-pku.cn/). This database was used to assess the link between DEGs expression and patient prognosis in multiple cancer types and drew the survival curve plot between them. Enter DEGs one by one into “Gene” and “LUAD” in “Datasets”. The prognosis-related genes could be got. Log-rank P-value <0.05 was considered statistically significant. DEGs with P < 0.05 were considered as genes that related to prognosis.

Moreover, receiver operating characteristic curves (ROC) were plotted to determine the sensitivity and specificity of these prognostic genes. Downloading clinical data, and analyzing the survival curve with the data through the TCGA database.

The ROC curves were drawn by the “pROC” package. The area covered under a curve is called the area under a curve (AUC). This is used to evaluate the performance of sensitivity and specificity. The higher the AUC, the better the effect by using the expression level to predict the survival time of cancer patients.

### Evaluation of the independent prognostic factor and survival analysis of the gene pair

Correlations between core gene expression level and the clinicopathological and molecular features were analyzed by the “Complex Heatmap”, “ggalluvial”, and “ggpubr” packages (21). According to the median expression level of core genes, LUAD patients were divided into high-expression and low-expression groups. In order to accurately study the relationship between gene expression and patient survival time, the relationship between the two groups of BTG2 and SerpinB5 and OS and progression free survival (PFS) were calculated by using the “survival” package. The clinical data and the gene expression RNA-Seq (HTSeq-FPKM) were downloaded from the TCGA dataset.

### Development and validation of the nomogram model

To establish the relationship between different clinical characteristics and patient survival, a prognosis model was constructed. Univariate and multivariate Cox regression analyses were used to determine whether core genes could be used as an independent prognostic factor in patients with LUAD without the influence of clinical characteristics.

The Cox regression model was constructed by the “RMS” (22) package and visualized the parameters related to the survival time of patients through nomogram. Nomogram is essentially a visual regression model. It sets the scoring criteria according to the regression coefficients of all independent variables and then gives the scoring values of each independent variable to calculate the total score of each patient. The conversion between occurrence probability and the prognosis were calculated to predict the survival time of each patient (22).

The concordance index (C-index) and a calibration curve plot were then used to evaluate the nomogram’s predictive accuracy and discriminative ability. The nomogram’s predictive accuracy was drawn by the “ggplot2” package. The x-axis represents the predicted survival rate of each patient, and the y-axis represents the actual survival rate of each patient. The correlations between core genes and co-expression genes were calculated by Pearson’s correlation analysis in the cBioPortal database (https://www.cbioportal.org/), and genes with a correlation coefficient (absolute value) more than 0.5 were selected.

### Enrichment in LUAD by GSEA and GO analysis

The GSEA is a computational analysis method used to judge whether an a priori-defined set of genes shows statistically significant differences between two biological states. In this study, the “clusterProfiler” package was used to perform GSEA between the high-expression and low-expression of core genes (23). Functional or pathway terms with adjusted P-values<0.05 and False Discovery Rate (FDR) q-value <0.25 were considered statistically significant. The GO analysis and KEGG analysis were also used to obtain the pathway that these genes may participate.

### Identification of potential mechanisms of lncRNA/miRNA/mRNA networks

In order to further study the possible mechanism of BTG2 and Serpinb5 in LUAD, the lncRNA/miRNA/mRNA network was used to reveal the mechanism. First, the miRNAs that were related to these two genes were screened through the “miRNA-mRNA” module in the StarBase v3.0 database (https://starbase.sysu.edu.cn/), and then the miRNAs that may be related to these two genes were obtained by the intersection of these two groups of genes. Then, the lncRNAs corresponding to the miRNAs were searched through the “miRNA-LncRNA” module. The screening condition was low stringency (>=1) in “CLIP Data”, and “Pan-Cancer” was ≥ 4 cancer types (24). The miRNAs and lncRNAs obtained above were used to build a network through Cytoscape.

### Infiltration patterns in the tumor microenvironment

The ESTIMATE algorithm (Estimation of Stromal and Immune cells in Malignant Tumors using Expression data) was applied to calculate the immune score, stromal score, estimate score, and tumor purity based on the expression level of mRNA of TCGA (25).

The ESTIMATE computational method in the “estimate” package was applied to calculate the “estimate score”, “immune score”, and “stromal score” in LUAD tissues. CIBERSORT computational method was used to compute cell components of the tissues. Twenty-two categories of TIICs (Tumor infiltrating immune cells), including plasma cells and natural killer cells were identified and the relative proportions were calculated by using the “CIBERSORT” package. Correlation analysis between different TIIC subpopulations was achieved by the “corrplot” package. The “vioplot” package was applied to visualize the TIICs between high-expression and low-expression groups. The association between the expression level of core genes and the TIICs was acquired by using “limma” “ggplot2” “ggpubr” and “ggExtra” packages.

Correlation analysis between different TIIC subpopulations was achieved by the “corrplot” package. For each tumor sample, the TMB was analyzed as the total count of somatic mutations (except silent mutations) detected in the tumor.

### Immunotherapy

Next, we further predicted the response that the LUAD patients treated with anti-PD-1 and anti-CTLA-4 immunotherapy. To better predict the response to the immune checkpoint inhibitors (ICIs), the immune cell and immunophenotype data were downloaded from The Cancer Immunome Atlas (TCIA) (https://tcia.at/home). The immunophenogram was used to predict anti-PD1/PD-L1 therapy response in LUAD. The immunophenogram was used to calculate the immunophenoscore (IPS) among four types (CTLA4 positive + PD-1 positive, CTLA4 negative + PD-1 negative, CTLA4 positive + PD-1 negative, CTLA4 negative + PD-1 positive, CTLA4 negative + PD-1 positive) from the TCGA database. The IPS scale ranged from 0 to 10. A high IPS predicts a good response to anti-PD-1/PD-L1 therapy. In addition, the correlation between expression level of the gene pair with the other immune checkpoint was also analyzed by Pearson’s correlation analysis and shown in a heatmap. The potential response of patients to immunotherapy was inferred by IPS and the tumor immune dysfunction and exclusion (TIDE) score. TIDE scores were calculated by the TIDE algorithm after normalizing the gene expression data (26). The tumor samples were divided into high-expression and low-expression according to the median value of expression level. Then, the TIDE score of the two groups were compared.

### Immunohistochemistry

The protein expression of core genes in both LUAD and normal tissues was obtained from the Human Protein Atlas database (HPA) (https://www.proteinatlas.org/), which is a program to map all the human proteins in cells, tissues and organs by using an integration of various omics technologies, including antibody-based imaging, mass spectrometry-based proteomics, transcriptomics and systems biology. In this study, the HPA database was used to analyze the protein expression level and performed immunohistochemistry (IHC) analysis of core genes between normal lung tissues and LUAD tissues.

### Molecular docking

To investigate the mechanism of the two genes binding with cisplatin, we made molecular docking between these two genes and cisplatin, respectively. We first obtained the molecular structure of the protein from the RCSB protein data bank (https://www.rcsb.org) and then the binding was obtained by Autodock software, which was used with default values for all parameters (27).

### Semi-quantitation RT-PCR

A total of 7 pairs of LUAD tissues and paracancerous tissues were collected from LUAD patients in SWMU hospital. The study was approved by the Ethical Committee of Southwest Medical University/Anhui University of Chinese Medicine, and all patients signed the informed consent form. All surgically removed samples were immediately transferred to liquid nitrogen and stored at -80°C until further research and analysis. The Use RNAsimple Total RNA Kit which was purchased from TIANGEN (Catalog No. DP419) was used to extract total RNA from the sample. The ReverTra Ace^®^ qRNA RT Master Mix which was purchased from TOYOBO (Code No. FSQ-201) was used to reversely transcribes RNA into cDNA. The procedure of reverse transcription was 37°C for 15min, 50°C for 5min, 98°C for 5min, and 4°C for holding. Then the cDNA was used as a template to prepare the PCR reaction solution. Veriti Thermal Cycler 96 Well (Applied Biosystems AB) was used for the amplification reaction. *ACTB* was used as an internal control. The sequences of the primers *ACTB* were: RT-ACTB-5: 5’-CTCTTCCAGCCTTCCTTCCT-3’ (forward primer), RT-ACTB-3: 5’-GTGGCCATCTGTGAGATCCT-3’ (reverse primer). The expected product size of *ACTB* was 510 bp. The sequences of the primers SERPINB5 were: RT- SERPINB5 -5: 5’- TTCCTTTTCCACGCATTTTC -3’ (forward primer), RT- SERPINB5 -3:5’- GTGGCCATCTGTGAGATCCT -3’ (reverse primer). The expected product size of SerpinB5 was 476 bp. The standard procedure of three-step PCR amplification was used: pre-denaturing at 95 °C for 30s, annealling at 60°C for 30s, and extending at 72°C for 30s. *ACTB* has 25 cycles and *SerpinB5* has 33 cycles (28–30).

### Statistical analysis

All statistical analyses and graphs were analyzed and displayed by R. P < 0.05 was considered to be statistically significant. P<0.05 is expressed by “*”; P<0.01 is expressed by “**”; P<0.001 is expressed by “***”.

## Results

### Identification of DEGs for LUAD that treated by cisplatin

**Figure 1:**
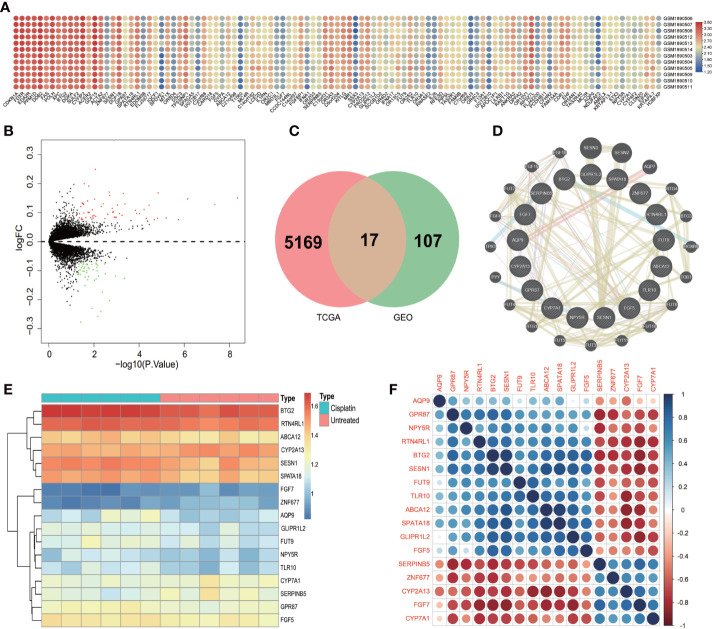
The differential expression genes. **(A)** Heatmap of the DEGs in GSE73302 according to the value of ∣log FC∣>1 and P <0.01. The green color indicates lower expression and red color indicates high expression. **(B)** The volcano plots visualize the DEGs in GSE73302. The red nodes represent upregulated genes while the blue nodes represent downregulated genes. **(C)** Common DEGs in GSE73302 and TCGA data sets. A total of 17 commons in the intersection of two gene set. **(D)** Protein–protein interaction network of differentially expressed genes and the related genes from the STRING database. **(E)** Heatmap of the 17 DEGs in GSE73302 according to the value of ∣logFC|>1 and P<0.01. The green color indicates low expression and red color indicates high expression. **(F)** A heat map shows the correlations of 17 DEGs in LUAD.

By unified processing of RNA-Seq data downloaded from the TCGA database, the mRNA gene expression levels in 59 normal samples were compared with 539 tumor samples and the results showed that 5169 genes were differentially expressed. There were 12 samples in the GSE73302 database, including 6 samples of the control group (lung cancer patients) and 6 samples of experimental groups (LUAD patients treated with cisplatin after 24h and 48h). The gene expression levels of the control group were compared with the experimental group and 107 genes were found to be differentially expressed. The change in gene expression level distribution in the GEO dataset can be seen in **Figures 1A, B**.

To obtain the DEGs that the LUAD patients were treated with cisplatin, the DEGs obtained from the TCGA dataset and DEGs obtained from the GEO dataset were intersected by the “Venn” package (**Figure 1C**). And a total of 17 DEGs were obtained. They were *ZNF677, TLR10, SPATA18, SESN1, SerpinB5, RTN4RL1, NPY5R, GPR87, GLIPR1L2, FUT9, FGF7, FGF5, CYP7A1, CYP2A13, BTG2, AQP9, ABCA12*. The changes in the expression level of 17 DEGs after being treated with cisplatin could be seen from the heatmap (**Figure 1E**).

### PPI analysis in LUAD

The PPI of the 17 DEGs network was established based on the STRING database with 14 edges and 17 nodes. The four genes with the most nodes were *SESN1, SerpinB5, GPR87, and BTG2.* There were four genes, including *ZNF677, TLR10, GLIPR1L2* and *FUT9*, that had no direct relationship with other genes in the PPI network (**Figure 1D**).

After analysis of the PPI network, 17 genes will affect each other. But the genes that how influenced each other was still unknown. Therefore, we need to explore the correlation between 17 genes. In this study, a heatmap was used to study the correlation (**Figure 1F**). As depicted in **Figure 1F**, the expression level of *SerpinB5* was negatively correlated with *BTG2*, *GDR87* and *SESN1*, and positively correlated with *FGFG7*. The expression level of *GDR87* was positively correlated with *BTG2* and *ABCA12*. The expression level of *BTG2* was positively correlated with *SPATA18* and *AQP9*. The expression level of *CYP2A13* was negatively correlated with *SPATA18* and positively correlated with *CYP7A1*. *NYP5R* was positively correlated with *RTN4RL1*. *FGF7* was negatively correlated with *FGF5* (**Figure 1F**).

### The mRNA expression of DEGs between LUAD tissue and Normal tissue.

**Figure 2:**
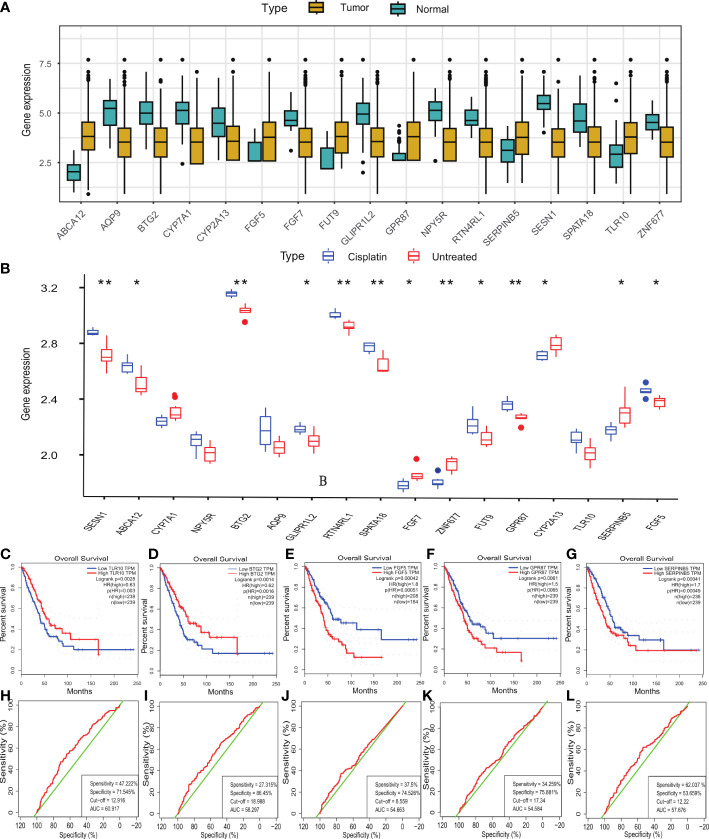
The mRNA expression level, survival analysis and ROC curve of DEGs. **(A)** The mRNA expression level of 17 DEGs in TCGA dataset. **(B)** The mRNA expression level of 17 DEGs in GSE73302 dataset. **(C-G)** The OS prognostic value of TLR10, BTG2, FGF5, GPR87, SerpinB5 in human cancer from GEPIA. **(H–L)** The ROC curve demonstrated the diagnostic value of TLR10, BTG2, FGF5, GPR87, SerpinB5 in LUAD patients. **(B)** *P <0.05, **P <0.01.

By comparing the mRNA expression level in the TCGA database, the result showed that compared with normal tissues, the genes with higher expression level of DEGs were *TLR10*, *SerpinB5*, *GPR87*, *FUT9*, *FGF5* and *ABCA12*. The genes with lower expression level were *ZNF677, SPATA18, SESN1, RTN4RL1, NPY5R, GLIPR1L2, FGF7, CYP7A1, AQP9*, CYP2A13 and *BTG2* (**Figure 2A**).

### Gene expression after cisplatin treatment

The DEGs with higher expression level after cisplatin treatment compared with the expression level of A549 were *TLR10, SPATA18, SESN1, RTN4RL1, NPY5R, GPR87, GLIPR1L2, FUT9, FGF5, BTG2, AQP9* and *ABCA12*. The genes with lower expression level after cisplatin treatment in LUAD were *ZNF677*, *SerpinB5*, *FGF7, CYP7A1 and CYP2A13* (**Figure 2B**). We sorted out the results of this part through a table

The mRNA expression level in normal lung tissue is expressed by “+”. “++” respect the mRNA expression level was increased in LUAD tissue, “-” respect the mRNA expression level was decreased in LUAD tissue. Compared with tumor group,there was more “+” when the mRNA level increased after treated with cisplatin. The specific changes of gene expression are shown in **Table 1**.

**Table 1:** The change of mRNA expression level in LUAD tissue, normal lung tissue and treated with Cisplatin.

Gene symbol	Expression			Gene symbol	Expression		
	Normal	Tumor	Cisplatin		Normal	Tumor	Cisplatin
ABCA12	+	++	+++	GPR87	+	++	+++
AQP9	+	–	+	NPY5R	+	–	+
BTG2	+	–	+	RTN4RL1	+	–	+
CYP7A1	+	–	–	SERPINB5	+	++	+
CYP7A2	+	++	+	SESN1	+	–	+
FGF5	+	++	+++	SPATA18	+	–	+
FGF7	+	–	–	TLR10	+	++	+++
FUT9	+	++	+++	ZNF677	+	–	–
GLIPR1L2	+	–	+				

The mRNA expression level in normal lung tissue is expressed by “+”. “++” respect the mRNA expression level was increased in LUAD tissue, “-” respect the mRNA expression level was decreased in LUAD tissue. Compared with tumor group,there was more “+” when the mRNA level increased after treated with cisplatin. “+++” respect the mRNA expression level was increased in LUAD tissue, which was increased compared with normal lung tissue, after treated with cisplatin.

From the above results, cisplatin could reduce the expression level of *CYP7A1, SerpinB5* which increased abnormally in LUAD and increase the mRNA expression level of *AQP9, BTG2, GLIPR1L2, NPY5R, RTN4RL1, SESN1, SPATA18* which decreased abnormally in LUAD. Therefore, the above genes may be the key genes of cisplatin in the treatment of LUAD. Next, the prognostic-related genes in DEGs were evaluated, and the results demonstrated that *TLR10, BTG2, FGF5, GPR87* and *SerpinB5* were significantly correlated with OS. Among them, the high-expression of *TLR10* and *BTG2* was significantly correlated with good OS. However, the low-expression of *FGF5, GPR87* and *SerpinB5* were significantly correlated with good OS (**Figures 2C–G**). Through the above research, *BTG2* and *SerpinB5* may play a therapeutic role in the treatment of LUAD with cisplatin and they were also mainly related to prognosis.

### The mRNA expression of *BTG2* and *SerpinB5* in pan-cancers and LUAD

**Figure 3:**
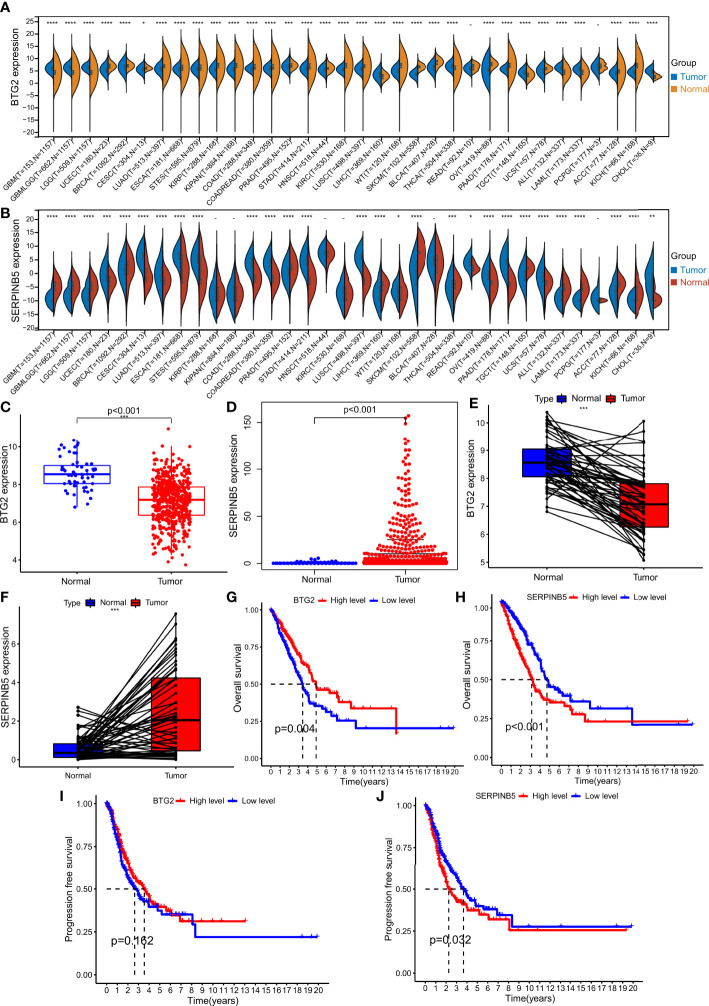
The expression levels in Pan cancer and LUAD and the survival analysis of BTG2 and SerpinB5. **(A)** BTG2 expression levels in multiple types of human cancers and adjacent normal tissues across TCGA (*P < 0.05, **P <0.01, ***P < 0.001). **(B)** SerpinB5 expression levels in multiple types of human cancers and adjacent normal tissues across TCGA. **(C, E)** The expression level of BTG2 in LUAD and normal lung tissues. BTG2 was more highly expressed in LUAD compared with normal lung tissues. **(D, F)** The expression level of SerpinB5 in LUAD and normal lung tissues. BTG2 was more highly expressed in LUAD compared with normal lung tissues. **(G–H)** The relationship between BTG2 and SerpinB5 expression and OS in LUAD patients. **(I-J)** The relationship between BTG2 and SerpinB5 expression and PFS in LUAD patients. **(A)** (*P < 0.05, **P <0.01, ***P < 0.001, ****P<0.0001, "-" respect P≥0.05).

*BT*G2 is differentially expressed between various cancers and normal tissues. The mRNA expression level in tissues of GBM, GBMLGG, LGG, BRCA, CESC, LIHC, THCA, TGCT, ALL, LAML, and CHOL was higher than that in normal tissues. There was no difference between PCPG, READ tumor tissues and normal tissues. The mRNA expression level of *BTG2* in tissues of UCEC, LUAD, ESCA, STES, KIRP, KIPAN, COAD, COADREAD, PRAD, STAD, HNSC, KIRC, LUSC, WT, SKCM, BLCA, PAAD, OV, UCS, PCPG, ACC, KICH was significantly different from that in normal tissues, and the mRNA expression level in tumor tissues was lower than that in normal tissues (**Figure 3A**).

By comparing the mRNA expression level of *SerpinB5* in tumor tissues with that in normal tissues, there was no difference in the mRNA expression of SerpinB5 between KIRP, KIPAN, HNSC, KIRC, BLCA and PCPG in normal tissues. The genes with higher mRNA expression level in tumor tissues than that in normal tissues were UCEC, CESC, LUAD, ESCA, STES, COAD, COADREAD, STAD, LUSC, WT, OV, PAAD, UCS and CHO. The genes with lower mRNA expression level in tumor tissues include GBM, GBMLGG, LGG, BRCA, PRAD, LIHC, SKCM, BLCA, REA, TGCT, ALL, LAML, ACC and KICH (**Figure 3B**).

Compared with normal lung tissues, *BTG2* mRNA expression level was lower in the tissues of LUAD, while the *SerpinB5* higher in LUAD tissues (**Figures 3C–F**).

### Survival analysis of *BTG2* and *SerpinB5*

The OS of patients with high *BTG2* expression was better than that of patients with low *BTG2* expression (P<0.05), and there was no significant difference in PFS between patients with high and low *BTG2* expression (P>0.05) (**Figures 3G, I**). The OS and PFS of patients with high *SerpinB5* expression were lower than those with low SerpinB5 expression (P<0.05) (**Figures 3H, J**).

### The relationship between *BTG2*, *SerpinB5* and the clinical characteristics of LUAD patients

**Figure 4:**
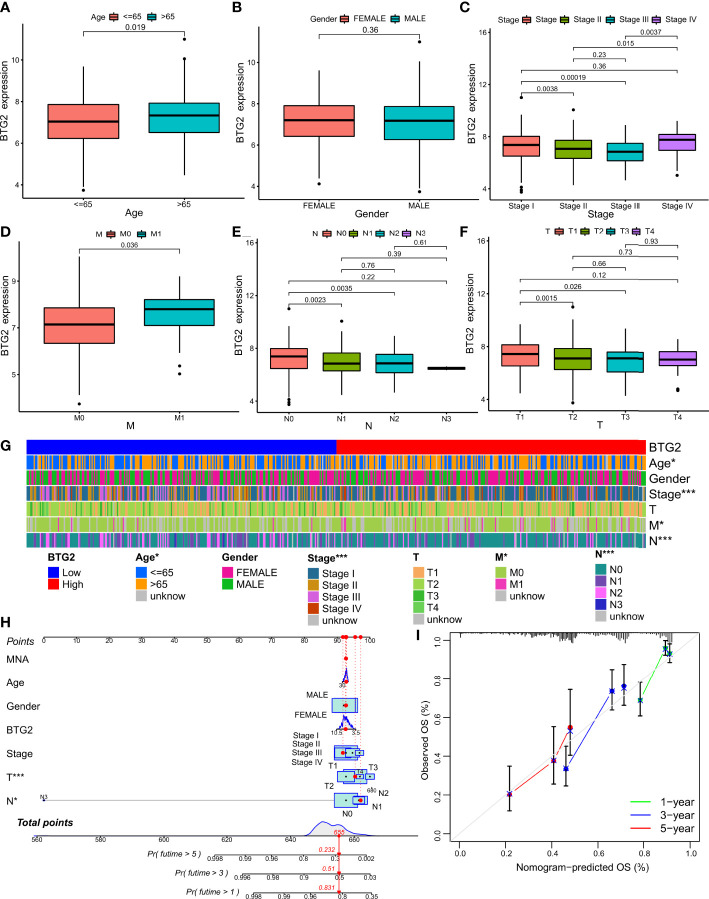
Relationship between the prognostic marker and clinicopathological factors of LUAD patients. **(A–F)** Correlation between BTG2 mRNA expression and clinical characteristics in patients with LUAD. P < 0.05 was considered significant. Age, Gender, Stage, M, N, T. **(G)** The heatmap shows the expression of the BTG2 and clinicopathological factors of LUAD patients in the high- and low-risk groups. **(H)** Construction BTG2-based nomogram for LUAD patients to predict OS. **(I)** The calibration curve and Hosmer–Lemeshow test of BTG2-based nomograms in the TCGA-LUAD cohort for OS. **(G)** (*P < 0.05, ***P < 0.001).

*BTG2* was differentially expressed in different N stages, M stages, pathological stages and different age groups (**Figure 4G**). The clinical baseline data was be shown in **Table 2**. There was no difference in the mRNA expression level of *BTG2* between different sexes (P>0.05) (**Figure 4B**), but it was differentially expressed between different age groups (P<0.019) (**Figure 4A**). The expression of *BTG2* in patients aged >=65 years was greater than that in patients aged <65 years (**Figure 4A**). It is also differentially expressed in different pathological stages. Stage I was differentially expressed with stage II and stage III respectively (P=0.0038, P=0.00019). Compared with Stage I, the gene expression of stage II and stage III are both down. There were significant differences in gene expression of *BTG2* between stage II, stage III and stage IV (P= 0.015, P= 0.0037). Compared with stage II and stage III, the gene expression of stage IV was relatively low (**Figure 4C**). It was differentially expressed between M0 and M1 (P=0.035), and the gene expression in M1 phase was lower than that in M0 (**Figure 4D**). N0 was differentially expressed with N1 and N2 (P=0.0023, P=0.0035), and N1 and N2 had lower gene expression than N0 (**Figure 4E**). It was differentially expressed among T1, T2 and T3 (P=0.0015, P=0.026), and the gene expression of T1 was higher than that of T2 and T3 (**Figure 4F**).

**Table 2:** The clinical baseline data.

Characteristic	All patients [cases (%)]	Characteristic	All patients [cases (%)]
**Gender**		**Clinical T stage**	
female	265 (54.1)	T1	168 (34.3)
male	225 (45.9)	T2	257 (52.4)
**Vital Status**		T3	44 (9)
Alive	312 (63.7)	T4	18 (3.7)
Dead	178 (36.3)	Others	3 (0.6)
**Age**	NA	**Clinical Stage**	
<65	218 (46.2)	Stage_I	266 (54.3)
>65	254 (53.8)	Stage_II	118 (24.1)
**Clinical N stage**		Stage_III	80 (16.3)
N0	316 (64.5)	Stage_IV	26 (5.3)
N1	91 (18.6)	**Clinical M stage**	
N2	70 (14.3)	M0	323 (66.3)
N3	2 (0.4)	M1	25 (5.1)
others	11 (2.2)	others	139 (28.5)

**Figure 5:**
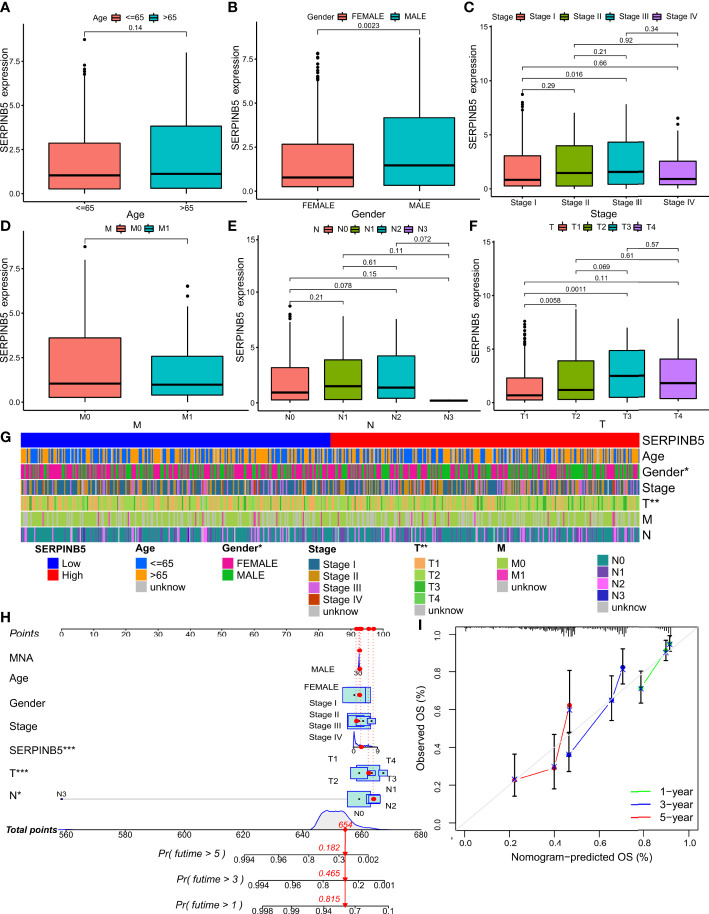
Relationship between the prognostic marker and clinicopathological factors of LUAD patients. **(A–F)** Correlation between SerpinB5 mRNA expression and clinical characteristics in patients with LUAD. P < 0.05 was considered significant. Age, Gender, Stage, M, N, T. **(G)** The heatmap shows the expression of the SerpinB5 and clinicopathological factors of LUAD patients in the high- and low-risk groups. **(H)** Construction SerpinB5-based nomogram for LUAD patients. predict OS. **(I)** The calibration curve and Hosmer–Lemeshow test of SerpinB5-based nomograms in the TCGA-LUAD cohort for OS. **(G)** (*P < 0.05, **P <0.01).

Overall, *SerpinB5* was differentially expressed in different T stages and different sexes (**Figure 5G**). The expression level of *SerpinB5* was not different in different age groups (P>0.05) (**Figure 5A**), but it was different between females and males (P=0.0023) (**Figure 5B**). Compared with female patients, the expression level of *SerpinB5* in male patients was higher (**Figure 5B**). In different pathological stages, *SerpinB5* was differentially expressed between stage I and stage III (P=0.016), and the gene expression of stage I was lower than that of stage III (**Figure 5C**). There was no difference in N stages and M stages (**Figures 5D, E**). It was differentially expressed in different T stages. The gene expression levels of T1, T2 and T3 were differentially expressed (P=0.0058, P=0.0011). Compared with T1, the gene expression levels of T2 and T3 were both higher (**Figure 5F**).

### The expression level of BTG2 and SerpinB5 impacted the prognosis of LUAD in patients with different clinicopathological status

**Figure 6:**
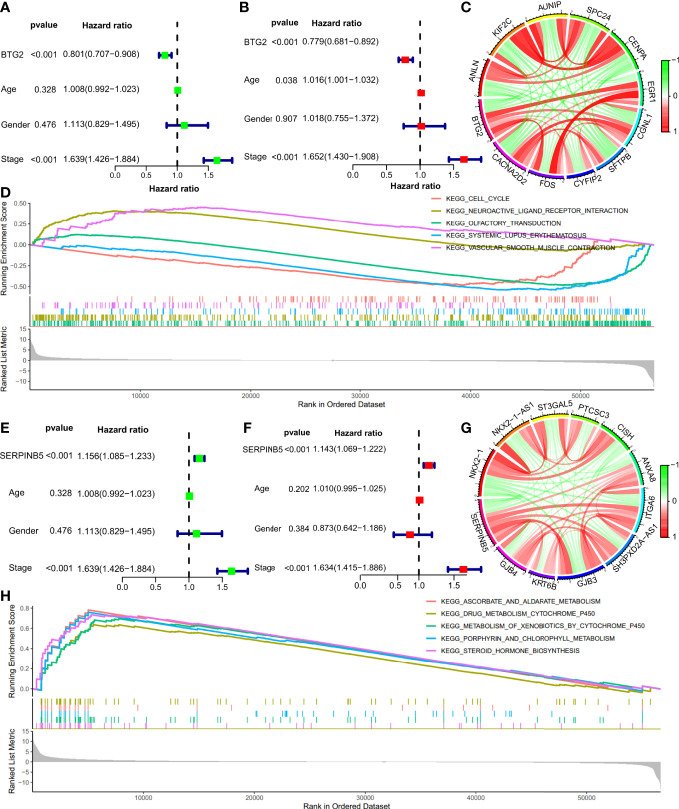
The Cox regression analyses, co-expression genes analyses, GSEA analyses of BTG2 and SerpinB5. **(A, B)** The univariate and multivariate Cox regression analyses of prognosis for BTG2 and clinicopathological factors. **(C)** The relationship of BTG2 and co-expression genes. The green color indicates negative correlation and red color indicates positive correlation. **(D)** The results of GSEA between high and low expression of BTG2 in LUAD patients. **(E, F)** The univariate and multivariate Cox regression analyses of prognosis for SerpinB5 and clinicopathological factors. **(G)** The relationship of BTG2 and co-expression genes. The green color indicates negative correlation and red color indicates positive correlation. **(H)** The results of GSEA between high and low expression of SerpinB5 in LUAD patients.

Cox regression was used to analyze the potential relationship between *BTG2*, *SerpinB5* and the OS of patients. Univariate Cox proportional hazards regression was used to assess the factors influencing OS. The results of the univariate Cox analysis suggested that BTG2 was a predictive factor for LUAD (HR: 0.801, CI: 0.701-0.908, P <0.001) (**Figure 6A**). Using the forest plot to demonstrate the results of the multivariate Cox analysis, the results showed that *BTG2* was an independent prognostic factor for the prognosis of patients with LUAD (HR: 0.779, CI: 0.681-0.892, P <0.001) (**Figure 6B**). These results suggest that *BTG2* can be used as a diagnostic and prognostic marker for LUAD.

The results of the univariate Cox analysis suggested that *SerpinB5* was a high-risk factor for LUAD (HR:1.156, CI:1.085-1.233, P <0.001) (**Figure 6E**). Using the forest plot to demonstrate the results of the multivariate Cox analysis, *SerpinB5* was an independent risk factor for the prognosis of patients with LUAD (HR: 1.143, CI: 1.069-1.222, P <0.001) (**Figure 6F**). These results suggest that SerpinB5 can be also used as a diagnostic and prognostic marker for LUAD.

### BTG2 and SerpinB5 co-expression in LUAD

In order to screen the core genes related to *BTG2* and *SerpinB5* and predict the regulatory relationship between genes, we constructed the co-expression network of *BTG2* and *SerpinB5*, respectively (**Figure 6C**). The results showed that *BTG2* has positive regulation with *CACNA2D2, FOS, CYFIP2, SFTPB, CGNL, EGR*. It has negative regulation with *CENPA, SPC24, AUNIP, KIF2C and ANLN*. And the results showed that *SerpinB5* has positive regulation with *GJB4*, *KRT6B, GJB4, SH3PXD2A-AS1, ITGA6, ANXA8*. It has negative regulation with *CISH, PTCSC3, ST3GAL5, NKX2-1-AS1 and NKX2-1* (**Figure 6G**).

### GSEA and GO Analysis of BTG2 and SerpinB5 in LUAD

In order to preliminarily explore the possible ways and pathways through which BTG2 and SerpinB5 function in the development of LUAD, the GSEA was used to perform enrichment analysis on BTG2 and SerpinB5. According to the p-value < 0.05, FDR < 0.05, significant enrichment pathways were screened. The results demonstrate that Aldosterone regulates sodium reabsorption, Neuroactivity, ligand receptor interaction and Vascular smooth muscle contraction were active when BTG2 was highly expressed. Olfactory conduction, Systemic lupus erythematosus were active when BTG2 was active at low BTG2 expression (**Figure 6D**).

The results demonstrate that Ascorbic acid and aldarate metabolism, Metabolism of xenobiotics by cytochrome P450, Porphyrin and chlorophyll metabolism, Retinol metabolism and Steroid hormone biosynthesis were active when SerpinB5 was highly expressed (**Figure 6H**).

**Figure 7:**
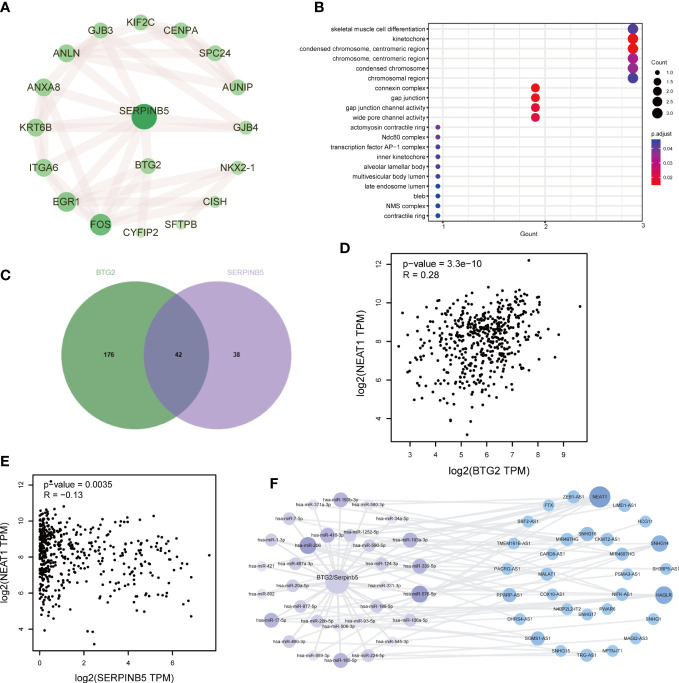
The potential mechanism of BTG2 and SerpinB5 in the LUAD. **(A)** PPI Network of BTG2, SerpinB5 and the co-expressed genes. **(B)** The GO enrichment analysis of BTG2, SerpinB5 and the co-expressed genes. **(C)** The counts of BTG2 related miRNAs, SerpinB5 related miRNAs and the intersection genes. **(D)** Correlation analysis of BTG2 and NEAT1 in LUAD. **(E)** Correlation analysis of SerpinB5 and NEAT1 in LUAD. **(F)** The LncRNA/miRNA/mRNA network.

The PPI network was made of genes related to BTG2 and SerpinB5, and the results show that FOS and EGR1 interact with many other genes in the PPI network (**Figure 7A**). By the GO analysis, the above genes were found to be mainly enriched in wide pore channel activity, gap junction channel activity, contractile ring, NMS complex and other functions. This may be the potential mechanism of these two genes (**Figure 7B**).

### Identification of lncRNA/miRNA/mRNA network

In order to study the mechanism, we also studied the potential lncRNA/miRNA/mRNA network. Searching for “BTG2” in the StarBase database, and a total of 218 miRNAs were obtained. Searching for “SerpinB5 “, and a total of 80 miRNAs were obtained. After the intersection of the two groups of miRNAs, 42 miRNAs were obtained (**Figure 7C**). Using these 42 miRNAs as keywords to search for relevant lncRNAs. These genes should be analyzed for correlation with BTG2 and SerpinB5 respectively, and a total of 31 lncRNAs were selected. The network results were shown in **Figures 7F**. NRAT1 was associated with more miRNAs and correlated with BTG2 and SerpinB5C (**Figures 7D, E**), so we speculate that these two genes may play a role through NRAT1.

### Relationship between mRNA expression of *BTG2* and *SerpinB5* and immune microenvironment and tumor mutational burden

**Figure 8:**
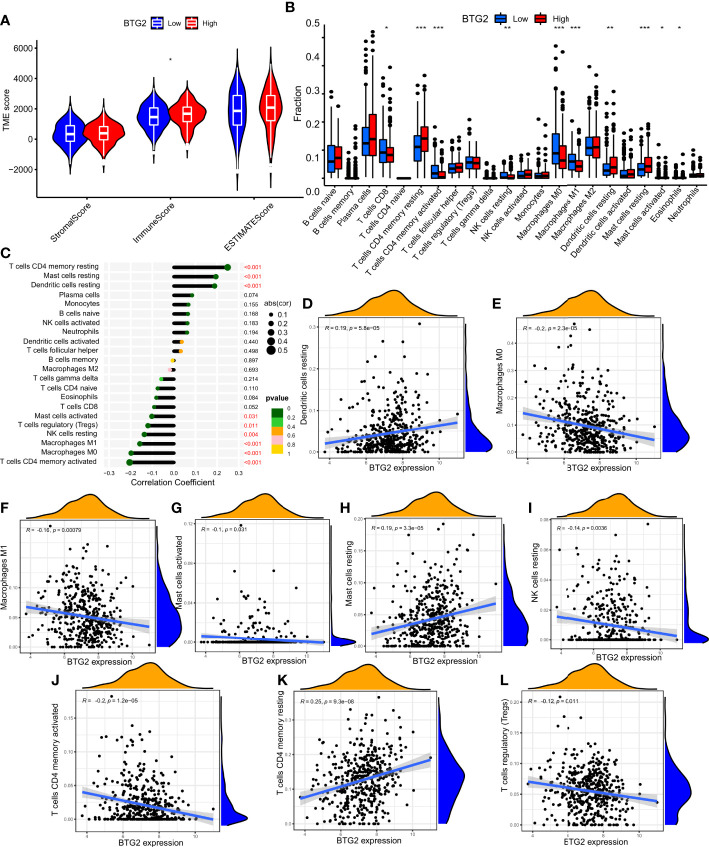
The expression of BTG2 was associated with immune infiltration in the LUAD microenvironment. **(A)** Composition of tumor microenvironment in high and low expression groups of BTG2. **(B)** Comparison of the infiltration of 22 leukocyte types between high and low BTG2 groups. **(C)** Correlation between the relative abundances of 22 immune cells and BTG2 expression level. **(D–L)** Correlation between BTG2 expression and immune cell infiltration in LUAD from TCGA sample. Dendritic cells resting, Macrophages M0, Macrophages M1, Mast cells activated, Mast cells resting, NK cells resting, T cells CD4 memory activated, T cells CD4 memory resting, T cells regulatory (Tregs). **(B)** [(*P < 0.05, **P <0.01, ***P < 0.001)].

**Figure 9:**
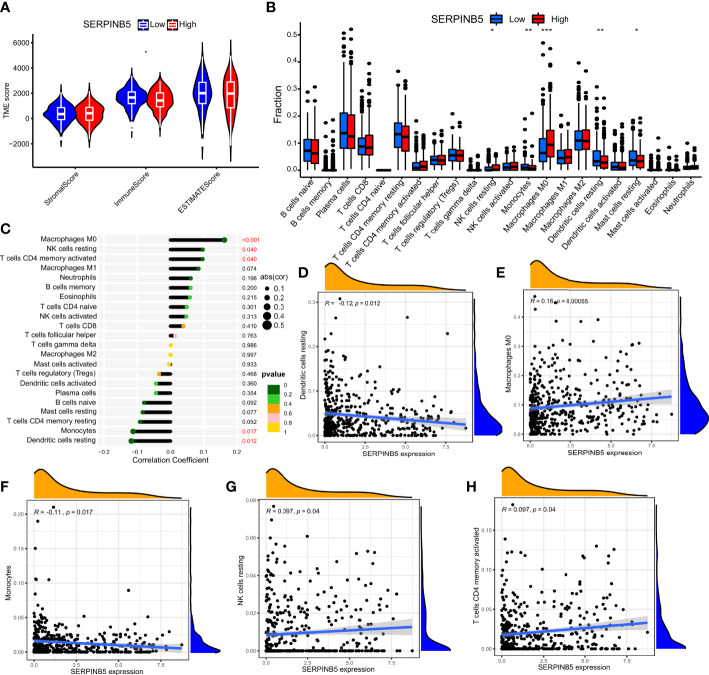
The expression of SerpinB5 was associated with immune infiltration in the LUAD microenvironment. **(A)** Composition of tumor microenvironment in high and low expression groups of SerpinB5. **(B)** Comparison of the infiltration of 22 leukocyte types between high and low SerpinB5 groups. **(C)** Correlation between the relative abundances of 22 immune cells and SerpinB5 expression level. **(D–H)** Correlation between BTG2 expression and immune cell infiltration in LUAD from TCGA sample. Dendritic cells resting, Macrophages M0, Monocytes, NK cells resting, T cells CD4 memory activated. **(B)** (*P < 0.05, **P <0.01, ***P < 0.001).

The immune microenvironment influences cancer progression by immune cells. To understand whether immune cells contribute to tumor growth, tumor immune cell infiltra1. There were significant differences in the number of immune cells between high and low expression groups of *BTG2* (P<0.05) and *SerpinB5* (P<0.05) (**Figures 8A**, **9A**). In the high-expression group of *BTG2* (**Figure 8A**) and the low-expression group of *SerpinB5* (**Figure 9A**), there were more immune cells in the immune microenvironment.

In order to further observe which immune cells are differentially expressed, the differentially expressed of *BTG2* in 22 immune cells was observed. The results showed that the *BTG2* in T cells CD8, T cells CD4 memory resetting, T cells CD4 memory activated, NK cells resting, Macrophages M0, Macrophages M1, Dendritic cells resting, Mast cells resting, Mast cells activated and Eosinophils were differentially expressed (**Figure 8B**). Besides, the correlation between gene expression and immune cells were also be studied (**Figure 8C**). The results suggest that mRNA expression level of *BTG2* were positively correlated with T cells CD4 memory resting (R = 0.25, p = 9.3e−08), Dendritic cells resting (R = 0.19, p = 5.8e−05), Mast cells resting (R = 0.19, p = 3.3e−05) and negatively correlated with Macrophages M1 (R = -0.16, p = 0.00079), T cells CD4 memory activated (R = -0.2, p = 1.2e−05), Macrophages M0 (R = -0.2, p = 2.3e−05), NK cells resting (R = - 0.14, p = 0.0036), Mast cells activated (R = - 0.1, p = 0.031), T cells regulatory (Tregs) (R = - 0.12, p = 0.011) (**Figures 8D–L**). The results showed that when the prognosis of patients with LUAD was poor, the expression level of *BTG2* was lower. Meanwhile, the immune cells which were positively related to the expression of *BTG2* may play an anti-tumor role. However, the immune cells negatively related to the expression of *BTG2* may play a role in promoting the occurrence and development of tumors.

**Figure 10:**
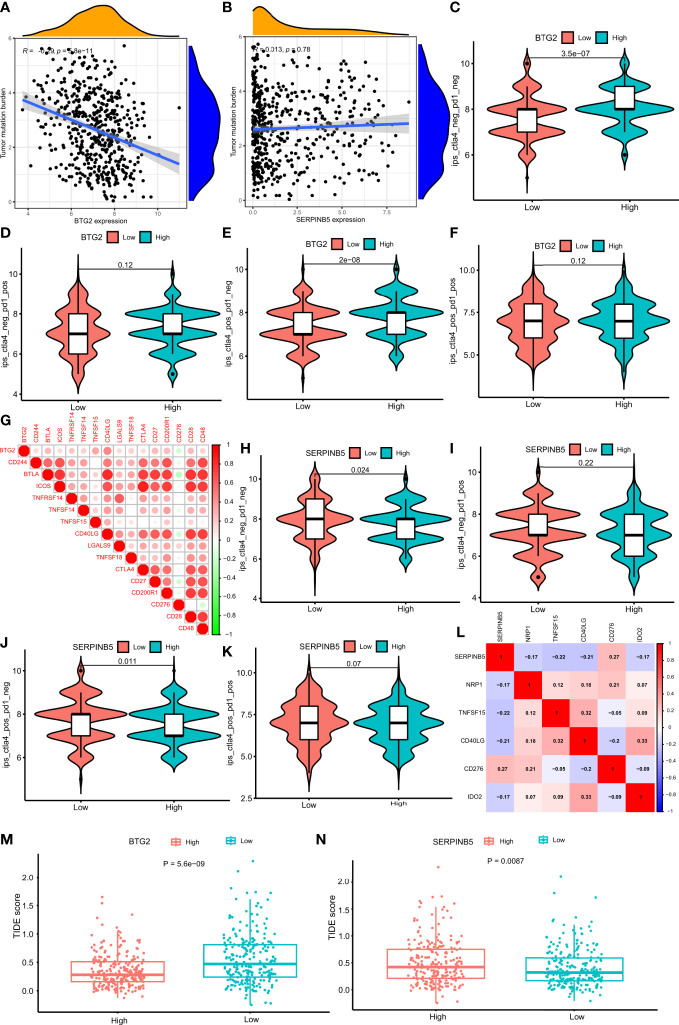
Comparison of the IPS in four groups and the relationship of the genes. **(A)** Correlation analysis of BTG2 expression and TMB in LUAD. **(B)** Correlation analysis of SerpinB5 expression and TMB in LUAD. **(C–F)** Comparison of the IPS between high- and low-expression groups of BTG2, IPS-CTLA4 negative + PD-1 negative, IPS-CTLA4 negative + PD-1 positive, IPS-CTLA4 positive + PD-1 negative, IPS-CTLA4 positive + PD-1 positive. **(G)** Correlations between BTG2 and Immune checkpoints associated with BTG2. Corr denotes Pearson correlation coefficient. The red nodes represent positive correlation with BTG2 while the green nodes represent negative correlation with BTG2. **(H–K)** Comparison of the IPS between high- and low-expression groups of SerpinB5, IPS-CTLA4 negative + PD-1 negative, IPS-CTLA4 negative + PD-1 positive, IPS-CTLA4 positive + PD-1 negative, IPS-CTLA4 positive + PD-1 positive. **(L)** Correlations between SerpinB5 and Immune checkpoints associated with the gene. Corr denotes Pearson correlation coefficient. The red nodes represent positive correlation with the gene while the green nodes represent negative correlation with the gene. **(M)** Boxplot representation of TIDE scores in the high-group versus low-group of BTG2 in TCGA LUAD cohort. **(N)** Boxplot representation of TIDE scores in the high-group versus low-group of SerpinB5 in TCGA LUAD cohort.

The two groups of *SerpinB5* were differentially expressed in T cells CD8, T cells CD4 memory resting, T cells CD4 memory activated, NK cells resting, Macrophages M0, Macrophages M1, Dendritic cells resting, Mast cells resting, Mast cells activated, Eosinophils among 22 immune cells (**Figures 9B, C**). The mRNA expression of *SerpinB5* were positively correlated with Macrophages M0 (R = 0.16, p = 0.00055), NK cells resting (R = 0.097, p = 0.04), T cells CD4 memory activated (R = 0.097, p = 0.04) (**Figures 9D, G, H**), and negatively correlated with Dendritic cells resting (R = -0.12, p = 0.012), Monocytes (R = - 0.11, p = 0.017) (**Figures 9E, F**). The mRNA expression level of *SerpinB5* was not correlated with TMB (P>0.05) (**Figure 10B**). The mRNA expression level of BTG2 was negatively correlated with TMB (R = - 0.29, P = 5.8e − 11) (**Figure 10A**).

The above results showed that when the gene expression of *BTG2* was low and the expression of *SerpinB5* was high, the prognosis of patients was poor when they were used as a gene pair as a prognostic marker. By analyzing the relationship between *BTG2*, *SerpinB5* and immune cells, the immune cells that were related to the changes of these two genes are Macrophages M0. At this time, the number of macrophages in the immune microenvironment increases, which indicates that the increase of Macrophages M0 may be a reason for the poor prognosis of LUAD patients.

### Relationship between *BTG2*, *SerpinB5* and immunotherapy

In order to study the relationship between mRNA expression and immunotherapy, the IPS produced by the high-expression and low-expression groups under the four treatment methods would be compared. The higher the IPS, the better the effect of immunotherapy. The results show that in CTLA4_ negative+PD-1_ Negative type and CTLA4_ positive + PD-1_ negative type, there was a significant difference in IPS between high-expression and low-expression of *BTG2* (P<0.05) (**Figures 10C, E**), and in CTLA4_ positive + PD-1_ Positive type and CTLA4_ negative+ PD-1_ positive type (**Figures 10D, F**), there was no significant difference in IPS between the two groups (P>0.05). Interestingly, in CTLA4_ negative+PD-1_ IPS of negative, *BTG2* high and low expression groups were higher than that of CTLA4_ positive + PD-1_ negative. The results showed that patients with high *BTG2* expression had a better therapeutic effect with the same immunotherapy. For patients with high expression, immunotherapy was better when CTLA-4 and PD-1 were inhibited at the same time.

By studying the relationship between the two groups of *SerpinB5* and immunotherapy methods, the results showed that in CTLA4_ negative+PD-1_ Negative and CTLA4_ positive + PD-1_ negative, IPS in the low-expression group was higher than that in high-expression group (P<0.005) (**Figures 10H, J**), and in CTLA4_ positive + PD-1_ Positive and CTLA4_ negative+ PD-1_ positive, there was no significant difference in IPS between high and low expression groups (**Figures 10I, K**). Interestingly, in CTLA4_ negative+PD-1_ Negative, IPS of high and low expression groups were higher than the IPS in CTLA4_ positive + PD-1_ negative. The results showed that patients with low *SerpinB5* expression had a better therapeutic effect with the same immunotherapy. For patients with low expression, CTLA4_ negative+PD-1_ Negative immunotherapy would be better.

In the TCGA LUAD cohort, the TIDE score of the high-expression group of *BTG2* was significantly lower than that of the low-expression group (**Figure 10M**). The TIDE score of the high-expression group of *SerpinB5* was significantly higher than that of the low-expression group (**Figure 10N**). By comparing the IPS and TIDE score of the high-expression group with the low-expression group of two genes, the potential immunotherapeutic effect of the high-expression group of *BTG2* would be better than that of the low-expression group, and the effect of the low-expression group of *SerpinB5* would be better than that of high-group.

### Correlation between BTG2 and SerpinB5 gene expression levels and immune checkpoint gene expression levels

*BTG2* was negatively correlated with immune checkpoint related gene *CD276*, and were positively correlated with *CD244*, *BTLA*, *ICOS*, *TNFRSF14*, *TNFSF14*, *TNFSF15*, *CD40LG*, *LGALS9*, *TNFSF18*, *CTLA4*, *CD27*, *CD200R1*, *CD28*, *CD48* (**Figure 10G**). Additionally, *SerpinB5* was negatively correlated with immune checkpoint-related genes *NRP1*, *TNFSF15*, *CD40LG*, *IDO2*, and positively correlated with *CD276* (**Figure 10L**). Both *BTG2* and *SerpinB5* were correlated with immune checkpoints *CD276* and *CD40LG*, while *BTG2* was negatively correlated with *CD276* and positively correlated with *CD40LG*. *SerpinB5* was positively correlated with *CD276* and negatively correlated with *CD40LG*. As a result, when *BTG2* was down-regulated and *SerpinB5* was up-regulated in LUAD, the expression of *CD276* increased and the expression of *CD40LG* decreased.

### Multiple methods for validation

**Figure 11:**
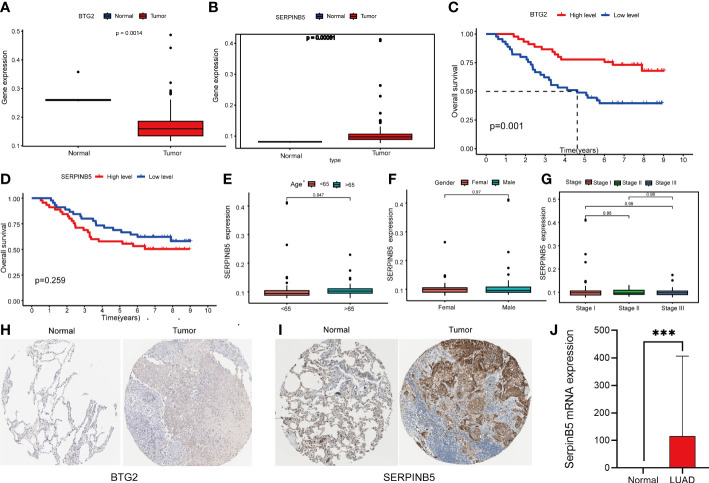
Analysis of BTG2 and SerpinB5 GSE11969 by dataset and Immunohistochemistry (IHC). **(A, B)** The expression level of *BTG2* and *SerpinB5* in LUAD and normal lung tissues in GSE11969 dataset. **(C, D)** The relationship between BTG2 and SerpinB5 expression and OS in LUAD patients. **(E–G)** Correlation between SerpinB5 mRNA expression and clinical characteristics in patients with LUAD. P< 0.05 was considered significant. Age, Gender, Stage. **(H, I)** Immunohistochemistry of BTG2 and SerpinB5 expression in LUAD tissues and corresponding normal tissues based on The Human Protein Atlas (HPA). **(J)** The results of SqRT-PCR of *SerpinB5*. Compared with paracancerous tissues, the mRAN expression were increasing in LUAD. **(J)** (***P < 0.001).

To verify the reliability of our analysis, we also investigated the changes in these two genes in other datasets. The GSE11969 database was downloaded, which was composed of 163 independent samples, including 158 lung samples and 5 normal lung tissue samples. We selected 90 LUAD patients from 158 patients and 5 normal patients as the study subjects. Differential analysis revealed that the two genes were differentially expressed in normal lung tissue and lung adenocarcinoma samples, with *BTG2* downregulated and *SerpinB5* upregulated compared with normal lung tissue, which is in agreement with the data we analyzed in the TCGA repository (**Figures 11A, B**).

In addition to these, we analyzed both genes in this dataset for survival analysis and correlation with clinical characteristics. The results showed that patients in the high expression group of *BTG2* had a better prognosis (**Figure 11C**). But showed no association of *SerpinB5* with patient outcome in this gene set. But there was no significant difference in *SerpinB5* by Survival analysis (**Figure 11D**).

Meanwhile, we analyzed the correlation between *SerpinB5* and clinical characteristics. The results showed that there were differences in mRNA expression level between different ages and different stages, but there was no difference between different genders (**Figures 11E–G**).

IHC staining images from HPA further validated the findings. IHC also indicated that SerpinB5 was remarkably overexpressed in the LUAD sample at the proteomic level, in comparison with the expression of SerpinB5 in normal Lung gland tissue (**Figure 11I**) and BTG2 was an inadequate expression in LUAD tissues (**Figure 11H**). The results of the analysis by the two methods agree with the results analyzed in the TCGA database.

In addition to the above studies, we also compared the mRNA expression of *SerpinB5* in LUAD with paracancerous tissues through qRT-PCR, and the results showed that the gene expression of *SerpinB5* was higher in LUAD tissues compared with paracancerous tissues, which was consistent with the results obtained by bioinformatics approach (**Figure 11J**). And the difference between *BTG2* was not significant. So we didn’t do too much research about *BTG2*.

### Molecular docking

**Figure 12:**
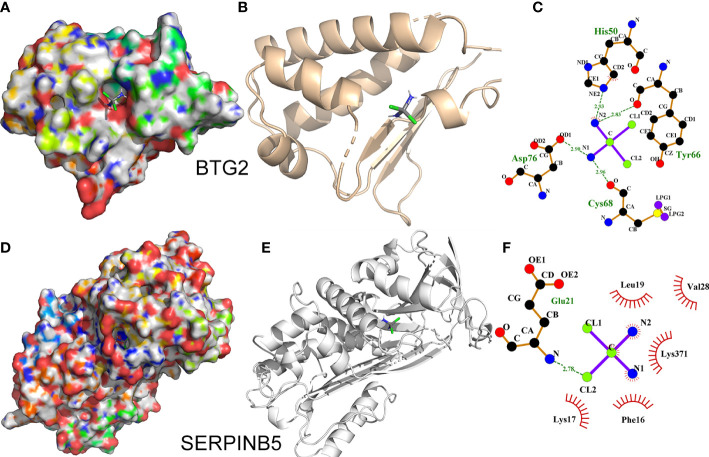
Interaction of BTG2 and SerpinB5 with cisplatin. **(A, D)** The binding mode of cisplatin to BTG2 and SerpinB5 in the active site. **(B, E)** Stereoview of binding mode for cisplatin with BTG2 and SerpinB5 in the binding site. **(C, F)** The detailed view of the 2-D ligand interaction among cisplatin with BTG2 and SerpinB5. The mRNA expression level in normal lung tissue is expressed by “+”. “++” respect the mRNA expression level was increased in LUAD tissue, “-” respect the mRNA expression level was decreased in LUAD tissue. Compared with tumor group,there was more “+” when the mRNA level increased after treated with cisplatin.

We simulated the binding situation of cisplatin with BTG2 and SerpinB5 by molecular docking, and the results showed that the binding affinities of BTG2 and SerpinB5 with cisplatin were mainly affected by hydrogen bonding and hydrophobic bonds (**Figures 12D–F**). Cisplatin forms H-bond networks with BTG2 in His50, Asp76, Tyr66 (**Figures 12D–F**). And cisplatin forms H-bond interactions with SerpinB5 in Glu21, while forms hydrophobic bonds in Leu19, Val28, Lys371, Phe16, Lys17 (**Figures 12D–F**).

## Discussion

NSCLC is the most common subtype of lung cancer, which can be divided into squamous cell carcinoma, large cell carcinoma and lung adenocarcinoma. Clinically, about 50% of patients were LUAD (31). Since most patients were diagnosed in the late stage of lung cancer, their 5-year survival time is difficult to exceed 15% after comprehensive treatment such as surgery, radiotherapy and chemotherapy (32). In recent years, the discovery of new molecular targets has promoted the development of new therapies such as targeted therapy and immunotherapy (25). For different treatment methods, there is an urgent need for stable and reliable prognostic biomarkers to identify subgroups with a high risk of death. Therefore, finding prognostic markers can effectively evaluate the survival probability of patients with LUAD and reasonably adjust the treatment methods.

Presently, in order to find appropriate tumor prognostic markers, we obtained DEGs in LUAD through bioinformatics technology. In addition, the cisplatin was used as the basic drug to study the genes whose gene expression changes when the drug acts. And the genes related to the OS of patients were also be studied. The genes that meet the above three conditions were regarded as genes that may become tumor prognostic markers. The results show that only BTG2 and SerpinB5 meet the above conditions. Compared with normal lung tissue, BTG2 was down-regulated in LUAD and *SerpinB5* was up-regulated in LUAD (**Figure 3**). After cisplatin treatment, cisplatin can increase the expression level of *BTG2* which was downregulated in LUAD compared with that in normal lung tissue, and decrease the expression level of *SerpinB5* which was upregulated in LUAD (**Figure 2A**) compared with that in normal lung tissue. At the same time, *BTG2* and *SerpinB5* were also related to the prognosis of patients. The prognosis was poor when *BTG2* was at low expression and poor when *SerpinB5* was at high expression (**Figure 3**). Therefore, we infer that *BTG2* and *SerpinB5* have the potential to become prognostic markers in patients with LUAD. Cox regression analysis showed that both of them were independent prognostic factors (**Figures 6**, **8**). Moreover, the nomogram also confirmed that when both were used as prognostic factors, their prediction accuracy was also high (**Figures 4**, **5**).

BTG2 was considered to be a tumor suppressor, which was highly expressed in a variety of normal tissues (33–36). It has been reported that BTG2 could play an anti-tumor role in a variety of ways. In the process of tumor occurrence and development, BTG2 played an important role in cell proliferation, differentiation, apoptosis and DNA damage repair. Wei found that overexpression of BTG2 can inhibit the proliferation and invasion of some tumors, including lung cancer cells (37). Zhang also found that BTG2 can promote or induce apoptosis of triple negative breast cancer cells and inhibit cell invasion (38).

SerpinB5 was first proposed as a tumor suppressor, and the mRNA expression level was downregulated in a variety of malignant tumors (39) compared with that in normal tissue. Some studies have found that SerpinB5 can inhibit tumor cell infiltration and metastasis, promote tumor cell apoptosis, and inhibit tumor vascular growth (40, 41). However, interestingly, our study found that *SerpinB5* expression level was up-regulated in LUAD (**Figures 3E, F**), and it may be used as a tumor inducer in the process of tumorigenesis. Lei found that SerpinB5 can promote the occurrence and development of gastric cancer in gastric cancer cell line HTB103 (42). However, there is no more in-depth study on SerpinB5 promoting the occurrence and development of gastric cancer. The results of this study showed that SerpinB5 has the potential to become an independent prognostic factor of LUAD (**Figure 6**), so it is necessary to further study the mechanism.

In addition to finding the relationship between the two genes, we reasoned the mechanism of the gene pair in LUAD from the perspective of lncRNA/miRNA/mRNA and finally deduced a pathway, which was NEAT1/miR-193b/SerpinB5 (BTG2) (**Figure 7**).

Besides, through correlation analysis, the mRNA expression of *BTG2* and *SerpinB5* was positively correlated (**Figure 1F**). This may be because both of them were p53 downstream regulatory genes. The recent study suggests that *BTG2* was originally identified as a p53‐inducible gene. Expression of BTG2 was significantly increased in response to DNA damage, and this increase was a consequence of p53 induction since the expression of a loss‐of‐function p53 mutant does not lead to BTG2 accumulation in this context (41). Meanwhile, SerpinB5 has also been reported to be the target gene of tumor suppressor gene p53. There was a p53 binding site in the promoter region of 84~112 nucleotides of the SerpinB5, and p53 can bind to this site to activate the SerpinB5 promoter and control its mRNA transcription. When wild-type p53 binds to the p53 binding site in the promoter region, it can stimulate histone acetylation and increase the accessibility of chromatin in the promoter region, thus activating p53 expression. On the contrary, mutant p53 will inhibit SerpinB5 expression (43). The positive correlation between BTG2 and SerpinB5 gene expression may be due to both being regulated by p53. However, in-depth research is needed on its specific relationship. By constructing the prognosis model, both *BTG2* and *SerpinB5* can be used to evaluate the 1-year, 3-year and 5-year survival rates of patients, and the accuracy of the model was high.

Through the study, it was found that *BTG2* was low expression and *SerpinB5* was high expression, and the prognosis of LUAD patients was poor. At this time, the active biological function of BTG2 was Olfactory conduction, Systemic lupus erythematosus. Among them, some studies have found that patients with systemic lupus erythematosus were easy to be associated with lung cancer, and there was a positive correlation between them (44). *BTG2* was low expression in LUAD and systemic lupus erythematosus.

The results of our study showed that when *BTG2* was low expression and *SerpinB5* was high expression in LUAD, the macrophage M0 in the tumor microenvironment increases during tumorigenesis. Resting macrophages can be polarized into a variety of subpopulations. Classically activated macrophages (M1) and alternatively activated macrophages (M2) are the two main subpopulations of macrophages (45). In the process of tumorigenesis, primary tumor cells can recruit macrophages to infiltrate the tumor microenvironment and become tumor associated macrophages (TAMs). Clinical studies have found that the proportion of TAMs in the primary focus of lung cancer patients was high, and the prognosis was poor (45). The study found that in the animal model of lung cancer, knocking out or blocking CSF1/CSF1R will significantly reduce the number of TAMs, proving that blocking the survival signal of macrophages was one of the effective ways to prevent and treat lung cancer (46). Results showed that BTG2 was negatively correlated with macrophage M0, and SerpinB5 was positively correlated with macrophage M0 (**Figures 8**, **9**). From the results of this study, when *BTG2* was low expression and *SerpinB5* was high expression, the macrophage infiltration level in tumor tissue increases, and the prognosis was poor. The results suggested that the increase of macrophages may be the main cause of poor prognosis in patients with LUAD (**Figure 8**, **9**). This research result was also consistent with the above clinical research report, showing that these two genes can not only be used as tumor prognostic factors, but also as drug targets to play a therapeutic role.

Additionally, in recent years, immunotherapy has gradually become a new anti-tumor therapy, in which ICIs was a common tumor immunotherapy in the clinic (42). The immune checkpoint was the regulator of the immune system, which can inhibit the function of T cells under normal circumstances (47). However, some tumors can regulate immune checkpoints to protect themselves from the attack of the host immune system and form immune escape (48). At present, the ICIs that have been listed mainly include CTLA-4 inhibitors and PD-1/PD-L1 inhibitors. Our results indicate that when PD-1/PD-L1 and CTLA-4 were inhibited, the immunogenicity in tumor tissue was higher. However, the immunogenicity of high-expression group of *BTG2* and the low-expression group of *SerpinB5* was also higher (**Figure 9**). In addition, the same results were obtained by comparing the TIDE scores of the high and low groups of these two genes. This indicates that the mRNA expression level of *BTG2* and *SerpinB5* may be detected to judge the effect of immunotherapy, making BTG2 and SerpinB5 may become prognostic biomarkers of immunotherapy.

Besides, both the two genes are related to *CD276* and *CD40* (**Figures 10G, L**), which were other immune checkpoints. Previous studies showed that CD276 could promote tumor immune escape, thus promoting the occurrence and development of tumors (49). However, CD40 was an inhibitory immune checkpoint, which can inhibit the occurrence and development of tumors (50). Isn conclusion, BTG2 and SerpinB5 were correlated with the above immune checkpoints, which may further prove that BTG2 and SerpinB5 have the potential as biomarkers of immunotherapy.

*BTG2* and *SerpinB5* were studied as a gene pair in our article to investigate their prognostic value in lung adenocarcinoma. This is the first time that the two genes were studied together to observe the prognostic value. Although there have been studies on the two genes separately, there was no one to report the combining of BTG2 and SerpinB5 (51–56). And we have also speculated the mechanism of how the gene pair influences the development of LUAD. By looking up the journal, we found that the two genes were p53-related genes (41, 43), and p53 was a key gene in tumor cell apoptosis (57, 58). It may also be the mechanism that this gene pair could become a prognostic marker for LUAD. In addition, we added the molecular dynamics simulation of BTG2 and SerpinB5 with cisplatin. Not only the molecular structure of the genes were displayed, but also the result demonstrated the genes could bind with cisplatin. And this is also the first time, the molecular structures of these two genes were presented in the article. At present, the common methods to find out prognostic markers were single gene analysis or constructing a prognostic model for prognostic analysis. Although the two methods are relatively common, the two methods are difficult to study the mechanism. However, it was found in our study that the gene pair were correlated about gene expression, and there may also be an interactive relationship in pathology. So it is easier to study the mechanism of the gene pair compared with other methods.

However, there are several limitations in this study. The present study mainly derived from public databases and was retrospective, but the sample size was small. Thus, to ensure greater reliability and representativeness of the findings and assumptions, the sample should be expanded for further research in the future. In addition, all data in this study were from public databases. Although the study included experimental verification, the sample size was small and the mechanism study could not be carried out.

## Conclusion

In conclusion, the expression of *BTG2* decreased and *SerpinB5* increased in LUAD. Downregulation *BTG2* gene expression in LUAD tissue could be upregulated, and the up-regulation *SerpinB5* in LUAD tissue compared with normal lung tissue could be down-regulated after being treated with cisplatin. The correlation analysis of gene expression between the two genes showed that the expression of *BTG2* was negatively correlated with the *SerpinB5*, they were both P53 downregulated genes, which gave us a hypothesis that they could be studied as a gene pair. the survival analysis show that when the *BTG2* gene expression was low and the *SerpinB5* was high, the patient’s prognosis was poor; Cox regression analysis showed that both *BTG2* and *SerpinB5* could be used as independent prognostic factors to evaluate the patient’s prognosis. Morever, the relationship between the two genes and the immune microenvironment was studied and showed that both of them are related to macrophages. The macrophages increased when the prognosis was poor, which may be a reason for the poor prognosis of LUAD patients. We also studied the response of these two genes to immunotherapy and that they also have the potential to become markers of immunotherapy. Take together, we proposed that *BTG2* and *SerpinB5* can be studied as a gene pair, but the common function of this gene pair has not been discussed in depth. In subsequent studies, it is necessary to conduct in-depth research and other experimental verification.

## Data availability statement

The data could be download at https://portal.gdc.cancer.gov/, https://www.ncbi.nlm.nih.gov/ and the code used during the current study are available from the corresponding author on reasonable request.

## Author contributions

JW and YY designed the study. JW performed data analysis and prepared figures. WY wrote the manuscript. CW, JC and WY did SqRT-PCR. RD, YL and YW scrutinized the data. JW and YY conceived the idea and coordinated the project. All authors contributed to the article and approved the submitted version.
